# Endoscopic closure of a rectal fistula using a cardiac septal defect occluder: the final redemption

**DOI:** 10.1055/a-2133-6176

**Published:** 2023-08-21

**Authors:** Renato Medas, Eduardo Rodrigues-Pinto, João Carlos Silva, Pedro Pereira, Guilherme Macedo

**Affiliations:** 1 Gastroenterology Department, Centro Hospitalar Universitário de São João, Porto, Portugal; 2 Faculty of Medicine of the University of Porto, Porto, Portugal; 3 Cardiology Department, Centro Hospitalar Universitário de São João, Porto, Portugal

An 84-year-old man with medical history of T1 bladder cancer underwent radical cystectomy, with subsequent complication of surgical site abscess. Abdominal computed tomography revealed free gas at the surgical site and a rectal wall defect suggestive of fistula. Despite conservative treatment, the patient had persistent penial and anal discharge and was referred for endoscopic closure.

Colonoscopy showed a 6-mm fistulous tract between the cystectomy surgical site and the rectal wall ( Fig. 1 ). Initial closure with 12/6 t over-the-scope (OTS) clips after margin epithelial ablation was attempted twice (second attempt with combined placement of a detachable snare below the OTS clip), 3 months apart ( Fig. 2 ). Despite initial technical and clinical success, the fistula recurred after spontaneous detachment of the OTS clips. Endoscopic internal drainage with a double-pigtail stent was also tried ( Fig. 3 ); however, the fistula persisted after distal spontaneous migration 2 months later. Given the persistence of the fistula, closure with a cardiac septal defect occluder (CSDO) was proposed.

**Fig. 1:**
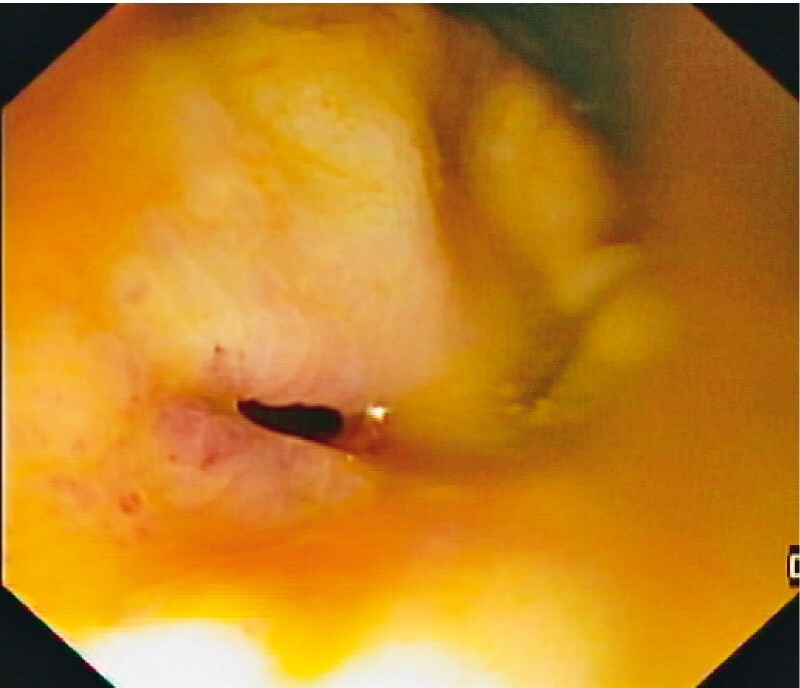
Rectal wall orifice (diameter 6 mm), corresponding to fistulous tract between the cystectomy surgical site and the anterior rectal wall seen at index colonoscopy.

**Fig. 2:**
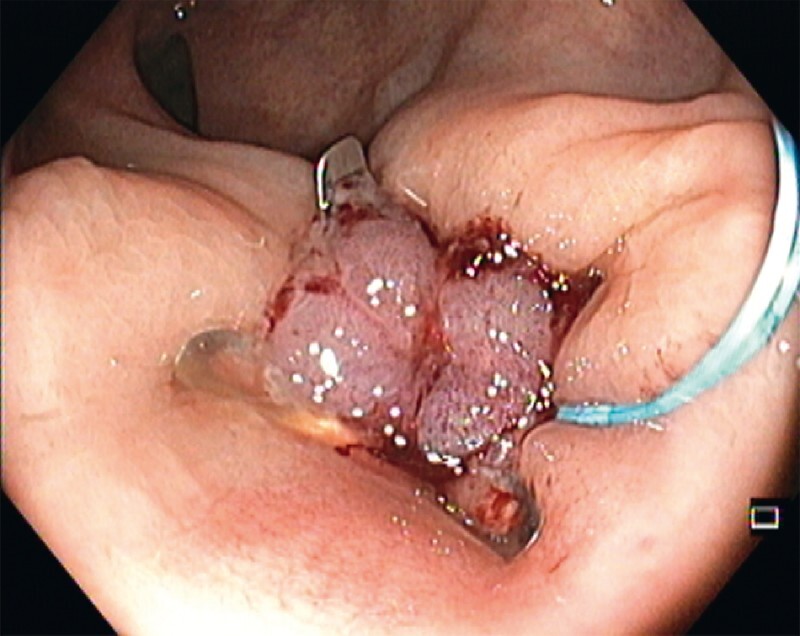
Combined use of a 12/6 t over-the-scope (OTS) clip and a detachable snare below the OTS clip after margin epithelial ablation.

**Fig. 3:**
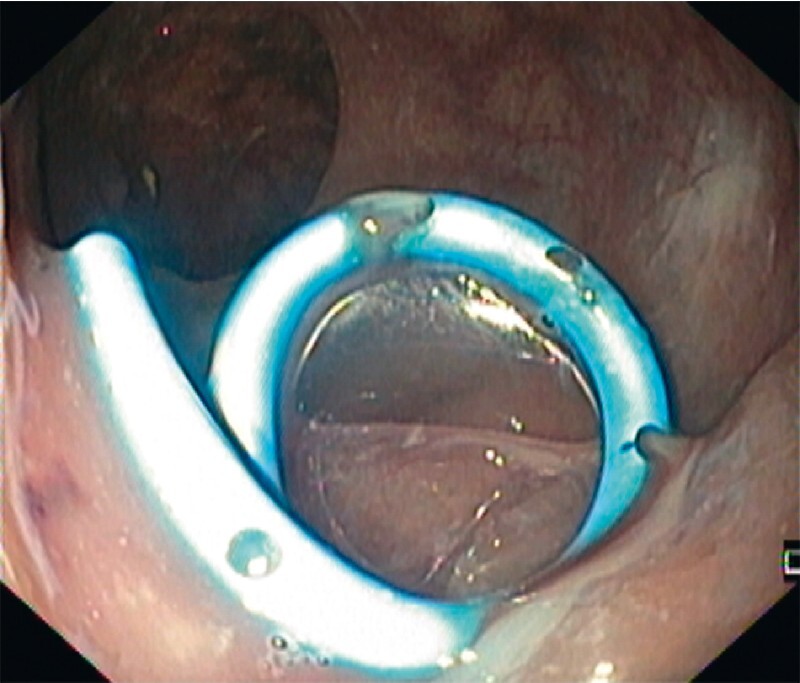
Endoscopic internal drainage using a 7-Fr 4-cm double-pigtail plastic stent.

After fluoroscopic characterization of the defect (surgical site 20 × 25 mm; fistulous tract length 4 mm; fistula orifice diameter 3 mm), a 16/4/12 mm CSDO was chosen ( Video 1 ). After placement of a 0.035-inch guidewire from the rectum into the surgical site, guided by a 5.4-Fr angiography catheter, a delivery sheath was advanced into the surgical site over the guidewire. After mounting the CSDO on the loading device, it was advanced inside the delivery sheath, and correctly deployed with the proximal flange in the surgical site and the distal flange in the rectum ( Fig. 4 , Fig. 5 ).

**Video 1** Use of a cardiac septal defect occluder for endoscopic closure of a fistula between the rectum and post-cystectomy surgical site.


**Fig. 4:**
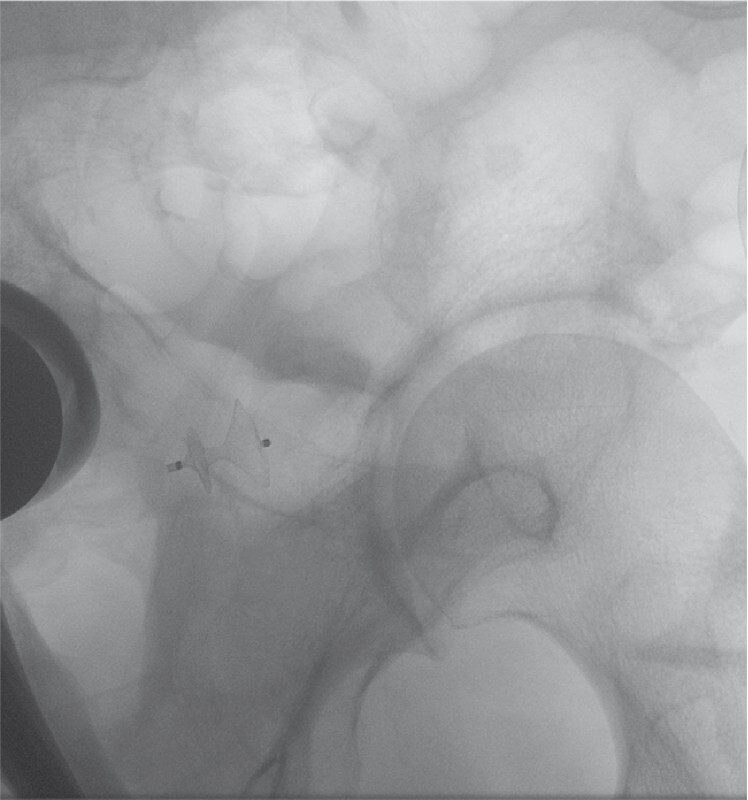
Fluoroscopic image of the cardiac septal defect occluder after deployment, with the proximal flange correctly opened in the surgical site and the distal flange in the rectum.

**Fig. 5:**
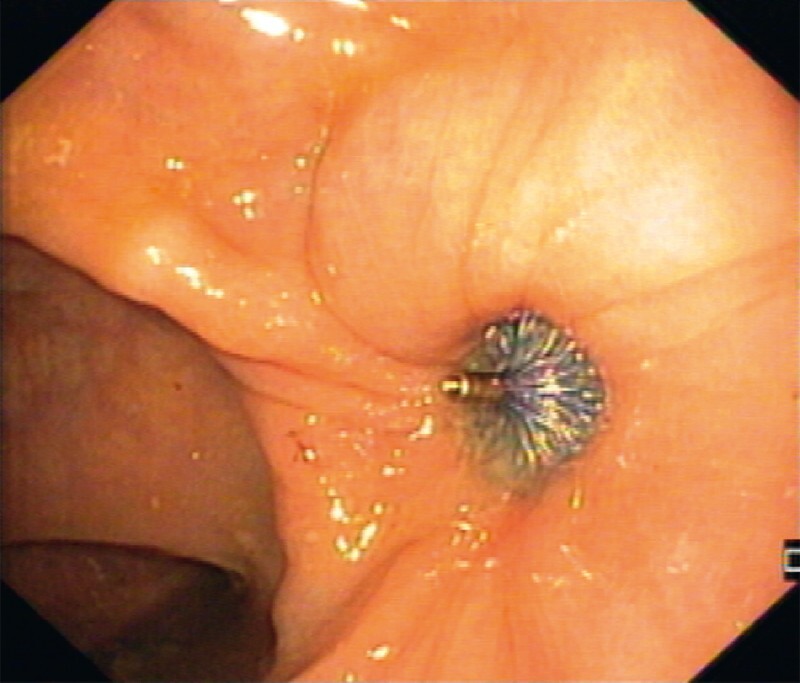
Endoscopic image of the cardiac septal defect occluder after deployment, with correct apposition between the device and the rectal wall.

The patient remains well, without clinical recurrence.

CSDO is an off-label device for closure of gastrointestinal fistulas and should be considered for chronic fistulas refractory to conventional endoscopic treatments 1 2 . To date, only four cases of lower gastrointestinal fistula (all rectovaginal) closure with CSDO have been reported 3 4 5 . To the best of our knowledge, this is the first case of endoscopic closure of a fistula between the rectum and surgical site.

Endoscopy_UCTN_Code_TTT_1AQ_2AG

## Notes

Endoscopy E-Videos

https://eref.thieme.de/e-videos
*E-Videos* is an open access online section of the journal *Endoscopy* , reporting on interesting cases and new techniques in gastroenterological endoscopy. All papers include a high-quality video and are published with a Creative Commons CC-BY license. Endoscopy E-Videos qualify for HINARI discounts and waivers and eligibility is automatically checked during the submission process. We grant 100% waivers to articles whose corresponding authors are based in Group A countries and 50% waivers to those who are based in Group B countries as classified by Research4Life (see: https://www.research4life.org/access/eligibility/ ). This section has its own submission website at https://mc.manuscriptcentral.com/e-videos
